# Fitness is positively associated with hippocampal formation subfield volumes in schizophrenia: a multiparametric magnetic resonance imaging study

**DOI:** 10.1038/s41398-022-02155-x

**Published:** 2022-09-16

**Authors:** Isabel Maurus, Lukas Roell, Daniel Keeser, Boris Papazov, Irina Papazova, Moritz Lembeck, Astrid Roeh, Elias Wagner, Dusan Hirjak, Berend Malchow, Birgit Ertl-Wagner, Sophia Stoecklein, Alkomiet Hasan, Andrea Schmitt, Andreas Meyer-Lindenberg, Peter Falkai

**Affiliations:** 1 Department of Psychiatry and Psychotherapy, University Hospital, LMU Munich, Munich, Germany; 2 NeuroImaging Core Unit Munich (NICUM), University Hospital LMU, Munich, Germany; 3 Department of Radiology, University Hospital, LMU Munich, Munich, Germany; 4 Department of Psychiatry, Psychotherapy and Psychosomatics of the University Augsburg, Medical Faculty, University of Augsburg, Bezirkskrankenhaus Augsburg, Augsburg, Germany; 5 Central Institute of Mental Health, Medical Faculty Mannheim, Heidelberg University, Mannheim, Germany; 6 Department of Psychiatry and Psychotherapy, University Hospital Göttingen, Göttingen, Germany; 7 Division of Neuroradiology, Department of Diagnostic Imaging, The Hospital for Sick Children, Toronto, ON Canada; 8 Department of Medical Imaging, University of Toronto, Toronto, ON Canada; 9 Laboratory of Neuroscience (LIM27), Institute of Psychiatry, University of Sao Paulo, São Paulo, Brazil; 10 Max Planck Institute of Psychiatry, Munich, Germany

**Keywords:** Hippocampus, Schizophrenia

## Abstract

Hippocampal formation (HF) volume loss is a well-established finding in schizophrenia, with select subfields, such as the cornu ammonis and dentate gyrus, being particularly vulnerable. These morphologic alterations are related to functional abnormalities and cognitive deficits, which are at the core of the insufficient recovery frequently seen in this illness. To counteract HF volume decline, exercise to improve aerobic fitness is considered as a promising intervention. However, the effects of aerobic fitness levels on HF subfields are not yet established in individuals with schizophrenia. Therefore, our study investigated potential associations between aerobic fitness and HF subfield structure, functional connectivity, and related cognitive impact in a multiparametric research design. In this cross-sectional study, 53 participants diagnosed with schizophrenia (33 men, 20 women; mean [SD] age, 37.4 [11.8] years) underwent brain structural and functional magnetic resonance imaging and assessments of aerobic fitness and verbal memory. Multivariate multiple linear regressions were performed to determine whether aerobic fitness was associated with HF subfield volumes and functional connections. In addition, we explored whether identified associations mediated verbal memory functioning. Significant positive associations between aerobic fitness levels and volumes were demonstrated for most HF subfields, with the strongest associations for the cornu ammonis, dentate gyrus, and subiculum. No significant associations were found for HF functional connectivity or mediation effects on verbal memory. Aerobic fitness may mitigate HF volume loss, especially in the subfields most affected in schizophrenia. This finding should be further investigated in longitudinal studies.

Clinical Trials Registration: The study on which the manuscript is based was registered in the International Clinical Trials Database, ClinicalTrials.gov (NCT number: NCT03466112) and in the German Clinical Trials Register (DRKS-ID: DRKS00009804).

## Introduction

The hippocampal formation (HF) is a complex structure that lies within the medial temporal lobe and comprises several subfields along its longitudinal axis [1]. These subfields show a high degree of specialization with regard to cytoarchitecture and include the hippocampus proper, the Cornu ammonis (CA; subfields CA 1–3), the dentate gyrus (DG) with its polymorph layer as CA4, the presubiculum, and the subiculum complex [2–4].

The HF plays a vital role in memory, navigation, and cognition and is considered to be essential for episodic memory because it provides a spatial and temporal framework for experiences [5]. The subfields within the HF contribute in distinct ways to memory formation. Rostral HF subfields such as the DG and CA2/3 are needed for the encoding of novel information, whereas caudal HF subfields such as the subiculum enable the retrieval of existing memories [6, 7]. As a crucial requirement for its cognitive and memory functions, the HF is involved in a tightly interconnected network that includes its subfields and extrahippocampal areas [1, 5]. The fimbria and fornix represent the efferent system of the HF and have projections to the rest of the brain [1].

In the clinical context, the HF is considered to be particularly susceptible to neurodegeneration in aging [8] and to be one of the key brain regions involved in the pathophysiology of psychiatric disorders, as was supported by the results of large-scale neuroimaging studies [2, 3]. HF atrophy is a well-established feature in schizophrenia [9], and pathological changes seem to occur in a subfield-specific manner [10]. Selected subfields, such as CA1, CA4, the DG, and the subiculum, have been suggested to be particularly vulnerable to volumetric reductions even at an early stage of schizophrenia [11, 12]. More widespread subregional and progressive global volume decline seems to occur with longer illness duration [13]. In addition to volume reductions, functional abnormalities of the HF have been demonstrated [13].

An association between HF morphologic and physiological abnormalities and cognitive deficits in individuals with schizophrenia has been consistently shown [14–18]. At the subfield level, negative correlations between CA1, CA2/3, CA4/DG, and subiculum volumes and cognitive functioning in individuals with schizophrenia have been demonstrated, whereby verbal memory seems to be particularly affected [19–21].

To counteract neurodegenerative effects and promote neuroregeneration, engagement in physical activity, and especially in exercise to improve aerobic fitness, is considered as a promising intervention [22]. Large-scale meta-analyses suggest that aerobic exercise may have the potential to prevent age-related decline in HF volume in the healthy population [23, 24], and positive correlations between aerobic fitness levels and HF volumes have been demonstrated repeatedly in structural magnetic resonance imaging (sMRI) studies in healthy individuals [22, 25].

Functional magnetic resonance imaging (fMRI) studies on the effects of exercise on the HF are few in number and have been conducted almost exclusively in healthy participants. The available evidence indicates that aerobic exercise and higher aerobic fitness levels may promote functional connectivity between the HF and several brain regions that are critical for cognitive functioning, whereby the strongest effects were found for the parahippocampal gyrus, middle frontal gyrus, and cingulate gyrus [26–28].

Across different imaging methods, studies in healthy populations that yielded positive associations between aerobic fitness and the HF also tended to demonstrate cognitive benefits [22, 29, 30], but results in individuals with schizophrenia remain scarce and equivocal [31]. A randomized controlled trial showed improvements in verbal memory after a 3-month aerobic exercise intervention [32] but no significant increase in HF or its subfield volumes in any of the study groups [33]. Almost all other schizophrenia‐related brain imaging studies on the effects of exercise focused on the overall HF volume and not its subfield volumes, despite the divergent functions of the subfields and their varying vulnerability in schizophrenia. Thus, it remains unclear whether potential associations are global or whether specific subfields may be subject to more localized effects. Furthermore, there is a lack of multiparametric MRI studies that examine not only HF morphology but also integrate resting-state fMRI (rs-fMRI) to provide further insights into the health of the HF circuitry. Studies aiming to elucidate the associations between exercise and fitness and the HF should also assess the related cognitive functions, a topic that has often been neglected [22].

To the best of our knowledge, our study in individuals with schizophrenia is the first to investigate the interrelations between aerobic fitness, HF subfield structure, HF functional connectivity, and verbal memory in a multiparametric research design. We hypothesize that there are regional specific, positive associations between aerobic fitness and HF subfield volumes. In addition, we hypothesize that aerobic fitness is positively related to functional connectivity within the HF subfields and between the HF and the parahippocampal gyrus, middle frontal gyrus, and cingulate gyrus and that potential associations between aerobic fitness and the HF mediate verbal memory functioning in schizophrenia.

## Methods and materials

The present study analyzed cross-sectional baseline (i.e., preintervention) data of participants in the Enhancing Schizophrenia Prevention and Recovery through Innovative Treatments (ESPRIT) C3 study.

Before participation in the study, participants provided written informed consent. All study procedures complied with the Declaration of Helsinki and were approved by the ethics committee of the Faculty of Medicine at the LMU Munich.

The C3 study, a multicenter, randomized, controlled, rater-blind clinical trial, investigated the effects of exercise on multiple levels in individuals with schizophrenia. In a two-armed parallel-group design, the study assigned participants either to an aerobic endurance training program on bicycle ergometers or a balance and tone training program as the control intervention.

To accurately measure the HF on its subfield level, a scanning resolution of less than 1 mm³ is recommended. Because this requirement was met only by the MRI scans performed at the C3 study center in Munich, the present study analyzed the data from this site.

### Study sample

At the Munich site, MRI was performed in 76 participants with schizophrenia and a valid baseline assessment of aerobic fitness data was available for 53 participants. For sMRI analysis, five participants had to be excluded because of a lack of a 3D magnetization prepared rapid gradient echo (MPRAGE) sequence, low image quality, or errors in the segmentation process. For rs-fMRI analyses, data from nine participants were excluded because of incomplete echo-planar imaging (EPI) sequences or insufficient data quality (see Supplemental Information, Quality control of MRI data and decisions on data exclusion). Therefore, data from 48 participants (29 men, 19 women; mean (SD) age, 37.4 (11.8) years) were included in the sMRI analysis, whereas data from 44 participants (27 men, 17 women; mean (SD) age, 37.7 [11.8] years) were included in the rs-fMRI analysis (for details, see Supplemental Information, Sample characteristics). All participants were diagnosed with schizophrenia in accordance with the DSM-IV criteria. Further inclusion and exclusion criteria, as well as other study details, can be found in Maurus et al. [34].

### Operationalization of aerobic fitness

While participants were exercising on a bicycle ergometer, a lactate threshold test was performed that provided assessed the lactate concentration in mmol/L in relation to the wattage in watts. Depending on the individual fitness level, lactate concentrations between 1.8 and 2.5 mmol/L represent the aerobic threshold, i.e., the level at which the lactate curve starts to rise exponentially [34, 35]. We identified the aerobic threshold for each participant and divided the achieved wattage at the aerobic threshold by body weight to ensure comparability of individuals. We used this value as the measure of aerobic fitness because it reflected each individual’s performance capability during aerobic exercise.

### MRI data acquisition and pre-processing

MRI data were acquired at the LMU Hospital Munich in a whole-body 3.0 Tesla MRI Scanner (Magnetom Skyra, Siemens Healthineers, Erlangen, Germany). The MRI scan consisted of one 3D T1-weighted MPRAGE sequence and two EPI sequences (see Supplemental Information, Scanning parameters) and was performed during rest. tRaw data files from the scanners were converted with dcm2niix software from DICOM to NIFTI files [36], which in turn were embedded into BIDS format [37].

Pre-processing of the structural T1-weighted images was performed with Freesurfer v7.2 (see Supplemental Information, Pre-processing of MRI data) and included motion correction and averaging [38], removal of non-brain tissue [39], automated Talairach transformation, segmentation of the subcortical white matter and gray matter volumes [40, 41], intensity normalization [42], tessellation of the boundary between gray and white matter, automated topology correction [43, 44], and surface deformation [45–47].

Pre-processing of the functional EPI images was performed with fMRIPrep [48] (see Supplemental Information, Pre-processing of MRI data). Independent Component Analysis-based Automatic Removal Of Motion Artifacts (ICA-AROMA) was employed to compute noise regressors [49]. The first 10 dummy scans of every pre-processed rs-fMRI file were removed. A Gaussian filter was used for smoothing (full-width at half-maximum = 6 mm), and the Nilearn v0.8.0 *clean_img* function was used for confound regression. In line with current findings on different denoising strategies, global signal, cerebrospinal fluid, white matter, and the extracted noise components from ICA-AROMA were regressed out from blood oxygenation level-dependent (BOLD) time series [50].

Quality control of sMRI and rs-fMRI raw images was performed with the automated software MRIQC [51]. The quality of the pre-processed sMRI images was evaluated with VisualQC v0.3.7.1 [52], and rs-fMRI data quality was monitored by evaluating various quality metrics after pre-processing, smoothing, and denoising (see Supplemental Information, Quality control of MRI data and decisions on data exclusion).

### MRI data post-processing

**Fig. 1:**
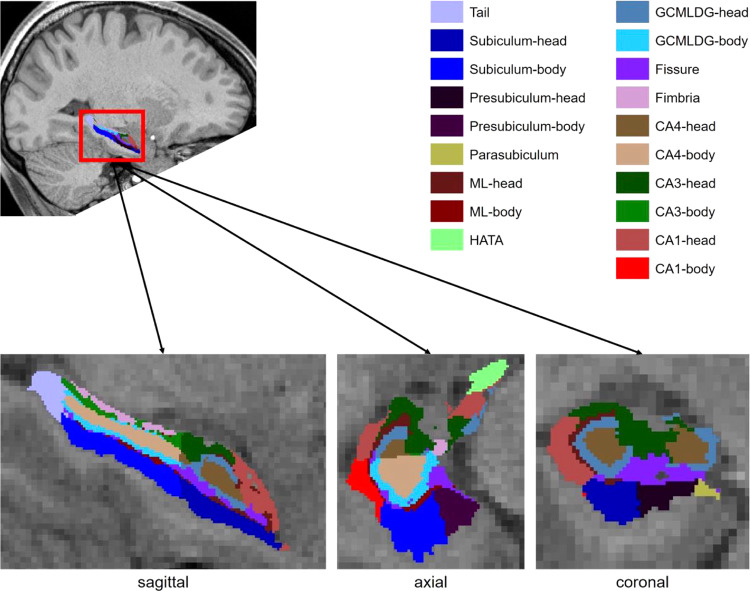
Hippocampal formation subfields. The hippocampal formation subfields segmented with Freesurfer v7.2 shown in different views. The table is based on Iglesias et al. [53] ML molecular layer, HATA hippocampal amygdala transition area, GCMLDG granule cell and molecular layer of the dentate gyrus, CA cornu ammonis.

For sMRI data analysis, the volumes of 38 HF subfields (19 per hemisphere) were computed from the T1-weighted 3D sequence by using the current extension of the hippocampal module of Freesurfer v7.2 [53]. The resulting volumes were concatenated in one file and corrected by the intracranial volume utilizing proportions method [54]. Figure 1 illustrates the segmented HF subfields and their anatomical location (note that CA2 is included in CA3). For more details on the definition of HF subfields, see Table 1.

**Table. 1:** Subfields of the hippocampal formation.

Name	Description
Tail	In the posterior part of the hippocampal formation
Subiculum	Divided into head and body; inferior part of the hippocampal formation
Presubiculum	Divided into head and body; inferior medial part of the hippocampal formation between the subiculum and parasubiculum (Brodmann 27)
Parasubiculum	Medial part of the hippocampal formation between the presubiculum and entorhinal cortex (Brodmann 49)
ML	Molecular layer: divided into head, body, and intermediate region, which contains parts of the subiculum or cornu ammonis (CA) fields
HATA	Hippocampus-amygdala transition area: superior medial part of the hippocampal formation
GCMLDG	Granule cell and molecular layer of the dentate gyrus: divided into head and body; between the molecular layer and CA4
Fissure	Anterior medial part of the hippocampal formation; white matter structure
Fimbria	Posterior superior part of the hippocampal formation; white matter structure
CA4	Cornu ammonis 4: inferior to the CA3 within the dentate gyrus
CA3	Cornu ammonis 3: superior to the dentate gyrus; includes CA2
CA1	Cornu ammonis 1: anterior lateral part of the hippocampal formation

*Note*. Description of the subfields of the hippocampal formation segmented with Freesurfer v7.2. For details on the segmentations, see Iglesias et al. [53].

For rs-fMRI data analysis, the *NiftiLabelsMasker* function from Nilearn v0.8.0 was used to extract the BOLD time series of seeds from the HF, parahippocampal gyrus, middle frontal gyrus, and cingulate gyrus as defined by the Brainnetome Atlas [55]. Functional connectivity between these participant-specific time series was computed with the *ConnectivityMeasure* function from Nilearn v0.8.0. Partial correlations were used to quantify functional connectivity, and the resulting correlation coefficients were converted to z-values with Fisher’s r-to-z transformation. For every participant, 166 functional connectivity measures were calculated that comprised functional connections between HF seeds, seeds from the HF and the parahippocampal gyrus, seeds from the HF and the middle frontal gyrus, and seeds from the HF and the cingulate gyrus. Table 2 provides a list of the examined seeds from the HF, parahippocampal gyrus, middle frontal gyrus, and cingulate gyrus based on the Brainnetome Atlas [55].

**Table. 2:** Seeds of interest from the Brainnetome Atlas.

Names of seeds from Brainnetome Atlas	Anatomical description
Hippocampus	
Hipp21	Rostral hippocampus
Hipp22	Caudal hippocampus
Parahippocampal gyrus	
PhG61	Rostral area of Brodmann 35/36
PhG62	Caudal area of Brodmann 35/36
PhG63	Lateral posterior parahippocampal gyrus
PhG64	Brodmann area 28/34 (entorhinal cortex)
PhG65	Temporal agranular insular cortex
PhG66	Medial posterior parahippocampal gyrus
Cingulate gyrus	
CG71	Dorsal area of Brodmann 23
CG72	Rostroventral area of Brodmann 24
CG73	Pregenual area of Brodmann 32
CG74	Ventral area of Brodmann 23
CG75	Caudodorsal area of Brodmann 24
CG76	Caudal area of Brodmann 24
CG77	Subgenual area of Brodmann 32
Middle frontal gyrus	
MFG71	Dorsal area of Brodmann 9/46
MFG 72	Inferior frontal junction
MFG 73	Brodmann area 46
MFG 74	Ventral area of Brodmann 9/46
MFG 75	Ventrolateral area of Brodmann 8
MFG 76	Ventrolateral of Brodmann 6
MFG 77	Lateral area of Brodmann 10

*Note*. The names of the examined seeds and their corresponding anatomical descriptions are taken from the Brainnetome Atlas [55].

### Cognitive data

Several cognitive tests were administered as part of the baseline data collection of the ESPRIT C3 study. To investigate potential associations between HF subfield volumes and HF-dependent cognitive domains, we chose to analyze data from the Verbal Learning and Memory Test (VLMT) [56] because a meta-analysis showed that HF volume is associated with verbal learning in schizophrenia [18] and a previous study found exercise-induced improvements in the VLMT in individuals with this disease [57].

In the VLMT, immediately after a list of 15 words had been read out loud, participants were asked to recall as many words as possible from the list. This process was repeated five times in succession, whereby data from the first trial were analyzed (VLMT-first). Thereafter, an interference list of 15 different words was read out loud, and participants were asked to recall the words from this list (VLMT-inter). After the interference trial, participants were asked to immediately recall as many words as possible from the initial target list without it being presented again (VLMT-sixth). After a 20-minute delay, participants were asked to recall as many words as possible from the target list again (VLMT-seventh). The numbers of correctly recalled words in VLMT-first and VLMT-inter were z-standardized and summarized to a short-term verbal memory score (VLMT-STM), and the numbers of correctly recalled words in VLMT-sixth and VLMT-seventh were averaged to a long-term verbal memory score (VLMT-LTM), similar to the approach used in a previous study [57].

### Statistical analyses

Rstudio v1.4.1717 based on R v4.1.2 was used for statistical data analyses [58, 59]. We detected outliers in the distributions of volumes, functional connectivity values, and VLMT scores (see Supplemental Information, Outlier detection). Age, sex, body mass index, disorder duration, education years, and chlorpromazine equivalents were defined as covariates, and chlorpromazine equivalents were computed by using the defined daily dose method [60]. Aerobic fitness values, volumes, VLMT scores, and all continuous covariables were z-standardized. After testing the corresponding pre-assumptions (see Supplemental Information, Tests of pre-assumptions of the multivariate multiple linear regression), two multivariate multiple linear regressions (MMLR) with aerobic fitness and the covariates as predictors and the volumes of 38 HF subfields and the 166 hippocampal functional connections as dependent variables were calculated with the *MVLM* package in R v4.1.2 [61]. If a significant main effect of aerobic fitness was found, mediation analyses with aerobic fitness as the independent variable, the hippocampal variables as mediators, and the VLMT-STM and -LTM scores as dependent variables were computed with the *mediation* package in R v4.1.2 [62]. Visualizations were performed with *ggplot2* package in R v4.1.2 [63].

## Results

### Relation between aerobic fitness and the volumes of the HF subfields

Table 3 summarizes the results from the MMLR analysis predicting the volumes of the HF subfields on the basis of aerobic fitness and the covariates. The overall MMLR model was not significant (R2pseudo = 0.196, T(7) = 1.393, *p* = 0.059), but aerobic fitness significantly predicted the volumes of the HF subfields (T(1) = 3.639, R2pseudo = 0.073, *p* = 0.003). The covariates were not significant.

**Table. 3:** Results from the multivariate multiple linear regression predicting volumes of the hippocampal formation subfields.

Predictor	T(df)	R²_pseudo_	*p*
Omnibus effect^1^	1.393(7)	0.196	0.059
Aerobic fitness	3.639(1)	0.073	0.003*
Age	1.203(1)	0.024	0.267
Sex	1.136(1)	0.023	0.305
Body mass index	1.538(1)	0.031	0.139
Education years	0.496(1)	0.010	0.875
Disorder duration	0.680(1)	0.012	0.692
Chlorpromazine equivalents	1.483(1)	0.030	0.155

^1^The omnibus effect represents the effect of the whole multivariate multiple linear regression model with *n* = 48.

Significant *p* values (<0.05) are written in bold and marked with an asterisk.

R²_pseudo_, effect size similar to the coefficient of determination; T(df), test statistics with numerator degrees of freedom.

**Fig. 2:**
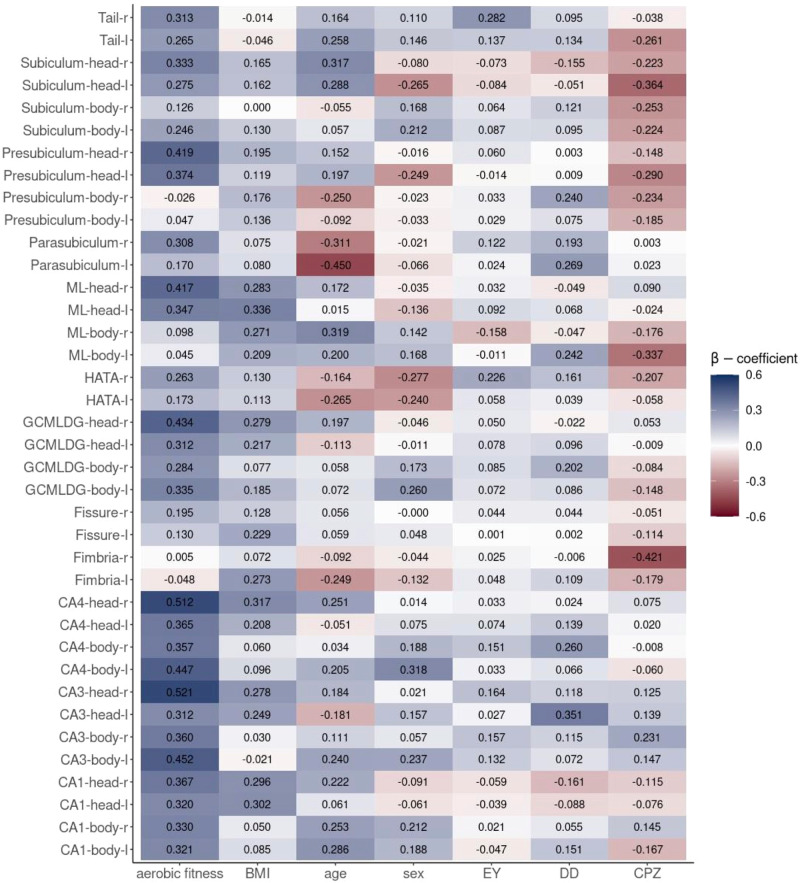
β-coefficients of aerobic fitness and the covariates predicting volumes of the hippocampal formation subfields. The x-axis shows the predictors of the multivariate multiple linear regression, and the y-axis, the 38 hippocampal formation subfields from both hemispheres. The heatmap is filled with the β-coefficients. Blue indicates a positive effect, red a negative effect. The darker the color, the stronger is the effect in the corresponding direction. BMI body mass index, CA cornu ammonis, CPZ chlorpromazine equivalents, DD disorder duration, EY education years, GCMLDG granule cell and molecular layer of the dentate gyrus HATA hippocampal amygdala transition area, l left hemisphere, ML molecular layer, r right hemisphere.

Figure 2 illustrates the regression coefficients β and indicates the size and direction of the effect of the predictors on volumes of each of the HF subfields. Aerobic fitness showed constant positive effects on all HF subfields apart from the bilateral fimbria and the bilateral bodies of the presubiculum and ML.

### Relation between aerobic fitness and static functional connectivity of the HF

Table 4 summarizes the results from the MMLR analysis predicting functional connectivity of seeds from the HF (Table 2) on the basis of aerobic fitness and the covariates. Neither the overall MMLR model (R2pseudo = 0.16, T(7) = 1.01, *p* = 0.442) nor the predictors were significant. The figures that visualize the regression β-coefficients indicating the size and direction of the effect of the predictors on the functional connectivity of seeds from the HF can be found in the Supplemental Information (Fig. S7.1 and S7.2).

**Table. 4:** Results from the multivariate multiple linear regression predicting functional connectivity of seeds from the hippocampal formation.

Predictor	T(df)	R²_pseudo_	*p*
Omnibus effect^a^	1.01(7)	0.16	0.442
Aerobic fitness	1.04(1)	0.02	0.399
Age	0.92(1)	0.02	0.597
Sex	0.86(1)	0.02	0.691
Body mass index	1.13(1)	0.03	0.274
Education years	1.14(1)	0.03	0.262
Disorder duration	1.05(1)	0.02	0.391
CPZ	0.87(1)	0.02	0.682

^a^The omnibus effect represents the effect of the whole MMLR model with *n* = 44.

R²_pseudo_ = effect size similar to the coefficient of determination; T(df), test statistics with numerator degrees of freedom.

### Mediation effects of volumes of the HF subfields

The HF subfields that showed a positive association with aerobic fitness were used as mediators between aerobic fitness and the VLMT-STM and -LTM scores, resulting in 64 separate mediation analyses. No significant mediation effects were observed after controlling for the false discovery rate. Regarding the VLMT-STM score, significant positive mediation effects (evaluated with uncorrected *p* values) were observed for the body of the left GCMLDG and the body of the left CA4 (Table 5). Regarding the VLMT-LTM score, a significant positive mediation effect (evaluated with uncorrected p values) was found for the body of the left GCMLDG, but a negative effect was found for the head of the left subiculum (Table 6).

**Table. 5:** Mediation between aerobic fitness, hippocampal formation subfield volumes, and Verbal Learning and Memory Test short-term memory scores.

Mediator	ACME	CI_low_	CI_high_	*p*	p_fdr_
Tail-r	0.061	−0.043	0.202	0.254	0.855
Tail-l	0.102	−0.006	0.255	0.060	0.480
Subiculum-head-r	0.004	−0.144	0.150	0.942	0.958
Subiculum-head-l	−0.053	−0.202	0.057	0.358	0.855
Subiculum-body-r	0.059	−0.021	0.193	0.186	0.792
Subiculum-body-l	0.046	−0.038	0.177	0.312	0.855
Presubiculum-head-r	−0.010	−0.173	0.158	0.840	0.958
Presubiculum-head-l	0.011	−0.156	0.159	0.848	0.958
Parasubiculum-r	−0.006	−0.125	0.099	0.926	0.958
Parasubiculum-l	−0.031	−0.140	0.033	0.434	0.855
ML-head-r	0.029	−0.110	0.179	0.692	0.886
ML-head-l	0.025	−0.126	0.183	0.684	0.886
HATA-r	0.122	−0.001	0.286	0.054	0.480
HATA-l	0.029	−0.084	0.159	0.592	0.886
GCMLDG-head-r	0.038	−0.120	0.216	0.648	0.886
GCMLDG-head-l	0.045	−0.082	0.191	0.448	0.855
GCMLDG-body-r	0.089	−0.015	0.235	0.106	0.678
GCMLDG-body-l	0.185	0.040	0.381	0.008*	0.256
Fissure-r	0.060	−0.027	0.189	0.198	0.792
Fissure-l	0.038	−0.061	0.171	0.448	0.855
CA4-head-r	0.059	−0.127	0.263	0.506	0.886
CA4-head-l	0.067	−0.067	0.228	0.336	0.855
CA4-body-r	0.093	−0.033	0.260	0.160	0.792
CA4-body-l	0.156	0.013	0.339	0.030*	0.480
CA3-head-r	0.068	−0.092	0.248	0.358	0.855
CA3-head-l	0.039	−0.056	0.176	0.454	0.855
CA3-body-r	−0.004	−0.106	0.099	0.958	0.958
CA3-body-l	−0.024	−0.164	0.106	0.662	0.886
CA1-head-r	0.043	−0.114	0.220	0.576	0.886
CA1-head-l	0.032	−0.126	0.211	0.666	0.886
CA1-body-r	0.007	−0.113	0.126	0.878	0.958
CA1-body-l	0.010	−0.112	0.139	0.896	0.958

*Note*. The table shows the results of 32 mediation analyses with *n* = 47. The hippocampal formation subfields that were associated with aerobic fitness were used as mediators between aerobic fitness and short-term verbal memory performance.

Significant *p* values (<0.05) are written in bold and marked with an asterisk.

*ACME* average causal mediation effect, *CI*_low_, lower confidence interval, *CI*_high_ higher confidence interval, *p* uncorrected p value, *p*_fdr_ fdr-corrected *p* value.

**Table. 6:** Mediation between aerobic fitness, hippocampal formation subfield volumes, and Verbal Learning and Memory Test long-term memory scores.

Mediator	ACME	CI_low_	CI_high_	*p*	p_fdr_
Tail-r	0.037	−0.089	0.190	0.520	0.776
Tail-l	0.043	−0.080	0.202	0.530	0.776
Subiculum-head-r	−0.060	−0.233	0.068	0.366	0.776
Subiculum-head-l	−0.128	−0.316	−0.009	0.020*	0.640
Subiculum-body-r	0.028	−0.043	0.133	0.486	0.776
Subiculum-body-l	0.027	−0.061	0.148	0.546	0.776
Presubiculum-head-r	−0.021	−0.207	0.145	0.790	0.832
Presubiculum-head-l	−0.051	−0.247	0.106	0.538	0.776
Parasubiculum-r	0.055	−0.057	0.198	0.346	0.776
Parasubiculum-l	−0.025	−0.132	0.047	0.558	0.776
ML-head-r	−0.071	−0.253	0.080	0.342	0.776
ML-head-l	−0.107	−0.297	0.034	0.130	0.776
HATA-r	0.052	−0.088	0.223	0.484	0.776
HATA-l	−0.023	−0.146	0.094	0.682	0.832
GCMLDG-head-r	−0.115	−0.308	0.052	0.186	0.776
GCMLDG-head-l	−0.041	−0.205	0.101	0.548	0.776
GCMLDG-body-r	0.062	−0.057	0.217	0.322	0.776
GCMLDG-body-l	0.141	0.007	0.320	0.040*	0.640
Fissure-r	0.014	−0.097	0.144	0.806	0.832
Fissure-l	0.028	−0.084	0.156	0.586	0.781
CA4-head-r	−0.101	−0.319	0.090	0.280	0.776
CA4-head-l	−0.020	−0.192	0.127	0.768	0.832
CA4-body-r	0.070	−0.065	0.248	0.330	0.776
CA4-body-l	0.130	−0.026	0.331	0.122	0.776
CA3-head-r	−0.107	−0.298	0.069	0.216	0.776
CA3-head-l	0.016	−0.099	0.150	0.780	0.832
CA3-body-r	−0.023	−0.161	0.073	0.704	0.832
CA3-body-l	0.023	−0.106	0.166	0.702	0.832
CA1-head-r	−0.124	−0.328	0.037	0.126	0.776
CA1-head-l	−0.086	−0.273	0.067	0.278	0.776
CA1-body-r	−0.055	−0.202	0.060	0.352	0.776
CA1-body-l	−0.007	−0.155	0.136	0.882	0.882

*Note*. The 32 mediation analyses with *n* = 47 are listed. The hippocampal formation subfields that were associated with aerobic fitness were used as mediators between aerobic fitness and long-term memory performance.

Significant *p* values (<0.05) are written in bold and marked with an asterisk.

*ACME* average causal mediation effect, *CI*_low_ lower confidence interval, *CI*_high_ higher confidence interval, *p* uncorrected *p* value, *p*_fdr_ fdr-corrected *p* value.

## Discussion

The main finding of our multi-parametric MRI-based study was that higher aerobic fitness levels were associated with higher volumes in the majority of HF subfields, especially in CA1 to CA4, the DG, and the subiculum. In our analyses of HF functional connectivity with the parahippocampal gyrus, no significant results were found for the middle frontal gyrus or cingulate gyrus. In addition, after controlling for the false discovery rate, no stable mediation effects were seen on the impact of relevant associations between aerobic fitness and HF subfield volume on verbal short- and long-term memory performance assessed with the VLMT.

Meta-analyses of randomized clinical trials concluded that aerobic exercise may prevent a decline in total HF volume, but significant results depended on the sample [23] and intervention characteristics [22–25, 64]. Older participants, who are more prone to neurodegeneration, benefitted the most from exercise interventions. Neurodegenerative processes have been suspected also in people with schizophrenia, but findings to date on the effect of exercise on HF volume remain scarce and inconclusive [23, 31, 65]. Within this debate, our study strengthens the notion of a positive relation between aerobic fitness and HF volume in schizophrenia.

The lack of previous cross-sectional studies on the interrelations between HF subfield volumes and aerobic fitness in individuals with schizophrenia complicates direct comparisons. At first glance, our results appear to differ from those of a single interventional study that considered the effects of exercise on the subfield level in participants with schizophrenia and observed no significant increase in the HF subfield volumes of the whole group after three months’ aerobic exercise training [33]. However, a post hoc analysis found a significant effect on CA3 and CA4 volume and functional recovery in about half of the patients under study [66]. Importantly, this trial evaluated the effects of a relatively short-term exercise intervention on HF subfield volumes, whereas our current cross-sectional study targeted the effect of general aerobic fitness, which depends on participants’ longer-term exercise involvement. In accordance with previous studies that demonstrated that longer exercise interventions yield more robust effects [22, 24], the divergent findings on the group level suggest that a greater increase in aerobic fitness could be a critical precondition for effects of exercise on HF volume.

A secondary analysis of the aforementioned study indicated that exercise-induced increases (or smaller decreases) of CA4 and DG volumes were modulated by participants’ individual polygenic risk scores for schizophrenia [67, 68] and by their cell-specific risk scores associated with oligodendrocyte precursor cells and radial glia [69]. When taken together, the former and present findings appear to indicate that aerobic fitness may be related to higher volumes of HF subfields in individuals with schizophrenia and that the underlying neuroplastic processes may be modulated by polygenic burden. In line with the literature on healthy individuals, the strongest effects were shown for CA1 to CA4, the DG, and the subiculum. These subfields are the most vulnerable to volumetric reductions, even at an early stage of schizophrenia [11, 12]. As a consequence, an association between aerobic fitness and total HF volume might be driven by regionally more specific associations within particular subfields. Thus, aerobic fitness might have the potential to mitigate schizophrenia-specific neurodegeneration or failed neuroregeneration, especially in the most affected HF subfields.

Considering the cognitive relevance of relations between aerobic fitness and the HF, our study does not support the hypothesis that aerobic fitness has a positive impact on verbal short- and long-term memory by increasing HF subfield volumes. In individuals with schizophrenia, our study was the first to examine these interrelations cross-sectionally, which again complicates direct comparisons with earlier research. In contrast to our findings, a previous interventional study in participants with schizophrenia reported that enhancements in aerobic fitness were related to increased total HF volume, which in turn was accompanied by improvement in short-term memory as assessed with the VLMT [57]. This interventional study considered only total HF volume, whereas we focused specifically on HF subfields and used a cross-sectional design. In addition, the two studies used different methods to compute HF volumes.

Some studies in healthy participants reported partially positive interrelations between aerobic fitness, HF subfield volumes (CA1, CA3/DG), and improved cognitive functioning [70–72], but associations tended to be weak. Future studies of HF subfield volumes need to include larger sample sizes for robust findings [13] because the positive interrelations between aerobic fitness and HF subfield volumes may hold true only for a subgroup of participants, analogous to the findings on plasticity with other kinds of interventions [73].

Concerning our rs-fMRI analysis, we did not detect associations between aerobic fitness and functional connectivity within the HF or between HF seeds and seeds from the parahippocampal gyrus, middle frontal gyrus, and cingulate gyrus (Table 2). In contrast, a previous study in participants with schizophrenia suggested that exercise-induced increases in hippocampal-prefrontal functional connectivity led to benefits in spatial memory [74], and another exploratory study in young adults at ultrahigh risk for psychosis demonstrated beneficial effects of aerobic exercise only on hippocampal-occipital functional connectivity [75]. However, research on the impact of aerobic fitness and exercise on HF functional connectivity in individuals with schizophrenia is very limited to date, and no comprehensive conclusions can be drawn yet. To our knowledge, our current study is the largest so far to address this research question. In healthy and other clinical populations, several widespread alterations in hippocampal functional connectivity related to aerobic fitness or exercise have been identified, but findings also are inconclusive [22, 28, 64, 76]. Again, differing study populations and methodological differences likely contribute to the discrepancies between findings.

To date, there is still a lack of multiparametric studies that not only examine hippocampal morphology but also attempt to provide further insights by integrating rs-fMRI data. We aimed to address this research gap and to assess not only the relation of aerobic fitness to HF subfield morphology but also the related cognitive impact. One of the major strengths of the present work is that it is one of the first studies in the field to focus on HF subfields with their divergent functions and varying vulnerability in schizophrenia, instead of investigating only total HF volume. In our study, we carefully considered covariables that might be associated with the variables of interest, and we could validate the results of our statistical model in the majority of the robustness checks (see Supplemental Information, Robustness checks of the MMLR). Furthermore, our data were obtained from participants with the same diagnosis and a relatively similar age; this homogeneity is important to avoid confounders, which tend to vary systematically between patient and control groups and within samples of different ages [77].

Our study has several limitations. First, we cannot rule out the presence of additional confounding factors. For example, in female study participants the menstrual cycle phase has been linked to changes in hippocampal connectivity [78] but was not assessed in our study. Second, our analyses required T1-weighted sequences at submillimeter voxel resolution, which were available only from a single study center. Even though our imaging protocol used a very high, state-of-the-art spatial resolution, the spatial resolution might still have been insufficient to assess all relevant anatomical variations [13]. Third, our study was cross-sectional in design, so caution should be exercised when interpreting the results for causality [79]. Fourth, the statistical power of the study was limited, increasing the risk that we missed potential small effects (see Supplemental Information, Post hoc power analyses). Therefore, the current results should be replicated in an independent sample. Last, for practical reasons genetic risk factors and subgroup characteristics were not accounted for in our study but might explain some of the heterogeneity observed [66, 68, 69, 80] and should be included in future investigations. In addition, future studies could use different HF-dependent tasks (for example, tasks targeting spatial memory) to evaluate whether cognitive functions other than verbal memory are more strongly associated with HF morphology.

Because of the key role of the hippocampus in memory formation and the changes in hippocampal volume seen in schizophrenia, we aimed to increase the knowledge about which parts of hippocampal structure and function are particularly interrelated with the effects of exercise. We showed that higher aerobic fitness levels were associated with higher volumes in the majority of HF subfields, with the strongest effects in CA1 to CA4, the DG, and the subiculum. These findings should be further investigated in longitudinal studies. Furthermore, despite the need for cautionary interpretation of our cross-sectional findings, we believe that they hold promise for a deeper understanding of the effects of exercise and the potential application of exercise as an add-on therapeutic approach in people with schizophrenia.

## Supplementary information

Supplemental material

## Data Availability

Imaging data, results from the quality control, the scripts for the whole analysis as well as demographic, physical, clinical and cognitive data files are published on OSF (Identifier: 10.17605/OSF.IO/TR3NX). Additional data will be made available upon request.
