# Supplementary material for: Fitness is positively associated with hippocampal formation subfield volumes in schizophrenia: a multiparametric magnetic resonance imaging study
Source: Transl Psychiatry. 2022 Sep 16;12:388. doi: 10.1038/s41398-022-02155-x (PMC9481539; doi:10.1038/s41398-022-02155-x)
Supplement: Supplementary file 1 — Supplemental material [file 41398_2022_2155_MOESM1_ESM.docx]

**Supplemental Information**

**Sample characteristics**

**Table S1**

Sample characteristics of the samples in the structural and functional magnetic resonance imaging analyses

| **Sample** | **N** | **Sex** | **Age, mean (SD), y** | **EY, mean (SD), y** | **DD, mean (SD), y** | **BMI, mean (SD)** | **CPZ, mean (SD)** |
| --- | --- | --- | --- | --- | --- | --- | --- |
|  |  |  |  |  |  |  |  |
| Structural MRI analysis | 48 | 29 men, 19 women | 37.38 (11.75) | 15.81 (4.17) | 10.49 (8.99) | 27.85 (4.56) | 390.76 (237.90) |
|  |  |  |  |  |  |  |  |
| Functional MRI analysis | 44 | 27 men, 17 women | 37.66 (11.79) | 15.89 (4.25) | 10.08 (8.49) | 27.86 (4.58) | 377.42 (212.29) |

BMI, body mass index; CPZ, chlorpromazine equivalents; DD, disorder duration; EY, years of education; MRI, magnetic resonance imaging; N, sample size

**Scanning parameters**

**Table S1**

Scanning parameters

| **Sequence** | **FoV** | **Resolution** | **TR** | **TE** | **TI** | **FA** | **Slices** | **Timepoints** |
| --- | --- | --- | --- | --- | --- | --- | --- | --- |
|  |  |  |  |  |  |  |  |  |
| MP-RAGE | 240 mm | 0.8 × 0.8 × 0.8 mm³ | 2060 ms | 2.17 ms | 1040 ms | 12° | 256 | - |
| EPI | 216 mm | 3.0 × 3.0 × 3.0 mm³ | 3000 ms | 30 ms | - | 85° | 45 | 124 |

*Note.* A 20-channel head coil was used.

EPI, echo planar imaging; FA, flip angle; FoV, field of view; MP-RAGE, T1-weighted magnetization prepared rapid gradient echo; resolution, voxel size; sequence, type of scanning sequence; slices, number of acquired slices; TE, echo time; TI, inversion time; TR, time of repetition

**Pre-processing of MRI data**

Pre-processing of the structural images was done with *recon-all* from Freesurfer and included motion correction and averaging [1], removal of non-brain tissue [2], automated Talairach transformation, segmentation of the subcortical white matter and grey matter volumes [3, 4], intensity normalization [5], tessellation of the boundary between grey and white matter, automated topology correction [6, 7], and surface deformation [8-10]. Further details on the Freesurfer pipeline can be found under http://surfer.nmr.mgh.harvard.edu/.

Pre-processing of the functional images was performed with fMRIPrep and comprised the following steps: T1-weighted (T1w) volumes were corrected for intensity non-uniformity with *N4BiasFieldCorrection v2.1.0* [11] and skull-stripped with *antsBrainExtraction.sh v2.1.0* (OASIS template) [12]; brain-extracted images were spatially normalized to the brain-extracted ICBM 152 Nonlinear Asymmetrical template version 2009c [13] by using nonlinear registration within the *antsRegistration* tool of ANTs v2.1.0 [14]; brain tissue segmentation was performed with *fast* from FSL v5.0.9 [15, 16]; resting-state fMRI data was slice-time corrected with *3dTshift* from AFNI v16.2.07 [17] and motion corrected with *mcflirt* from FSL v5.0.9 [16, 18]; distortion correction was performed by co-registering the fMRI image to the same-individual T1w image with intensity inversion [19, 20] constrained by an average field map template [21] implemented in *antsRegistration* [14]; co-registration to the corresponding T1w image by boundary-based registration [22] with twelve degrees of freedom was executed with *flirt* from FSL v5.0.9 [16, 18, 23]; motion-correcting transformations, field distortion-correcting warp, BOLD-to-T1w transformation, and T1w-to-template (MNI) warp were administered in one step with *antsApplyTransforms* from ANTs v2.1.0 [12]; frame-wise displacement (FD) [24] was computed for every functional run by using the implementation in Nipype [25]; and independent Component Analysis-based Automatic Removal Of Motion Artifacts (ICA-AROMA) was used to extract aggressive noise regressors [26]. For more details on the fMRIPrep pipeline, see <https://fmriprep.readthedocs.io/en/stable/workflows.html>.

The commands and results from Freesurfer and fMRIPrep will be published on OSF.

**Quality control of MRI data and decisions on data exclusion**

Quality control was performed at four stages of the analysis: First, structural and functional raw data were inspected with the automated quality control software MRIQC [27]. Second, structural and functional images were quality controlled again after pre-processing with the software VisualQC [28] for the structural images and the corresponding quality metrics (Framewise Displacement and DVARS) from fMRIPrep [29] for the functional images; regarding the latter, the signal-to-noise ratio (SNR) was also evaluated with *fslmaths* from FSL v 6.0.4 [16]. Third, the SNR of the functional data was computed again after image smoothing. And fourth, after denoising of functional images, their quality was checked by examining the correlations between the individual-specific denoised timeseries and the framewise displacement time series and between the mean framewise displacement values and the functional connectivity values across the whole sample. All commands, scripts, and documentation sheets used for quality control at the four different stages and the results from MRIQC and VisualQC will be published on OSF.

Reliable fitness data were available for 53 participants who underwent MRI scans. Of those 53 individuals, the following five were excluded from the structural MRI analysis because of missing scanning sequences, insufficient image quality, or errors during the segmentation process: MLxSCZ006, MLxSCZ007, MLxSCZ060, MLxSCZ07,3 and MLxSCZ076. Therefore, a total of 48 participants were included in the final structural analysis. Furthermore, the following nine individuals were excluded from the fMRI analysis because sequences were either missing or of low quality: MLxSCZ006, MLxSCZ011, MLxSCZ012, MLxSCZ031, MLxSCZ060, MLxSCZ066, MLxSCZ069, MLxSCZ073, and MLxSCZ076. Consequently, fMRI data of 44 participants were included in the analysis.

**Outlier detection**

Figures S5.1, S5.2, S5.3, and S5.4 depict the distributions of the z-standardized memory scores, volumes, and functional connectivity values. Z-scores higher than 3 or lower than -3 were defined as outliers. Outliers were present only in the volume data (Figure S4.2). In particular, the participants MLxSCZ047 and MLxSCZ048 had rather high volumes in the body of the right CA3 and the right fimbria, respectively, and participant MLxSCZ071 had low volumes in the body of the right CA4 and the right tail. However, the data quality of the corresponding images was sufficient, and their segmentations worked well as indicated by the quality control with VisualQC. Therefore, they were included in the final analysis.

**Figure S5.1**

Distributions of the short- and long-term memory scores in the Verbal Learning Memory Test


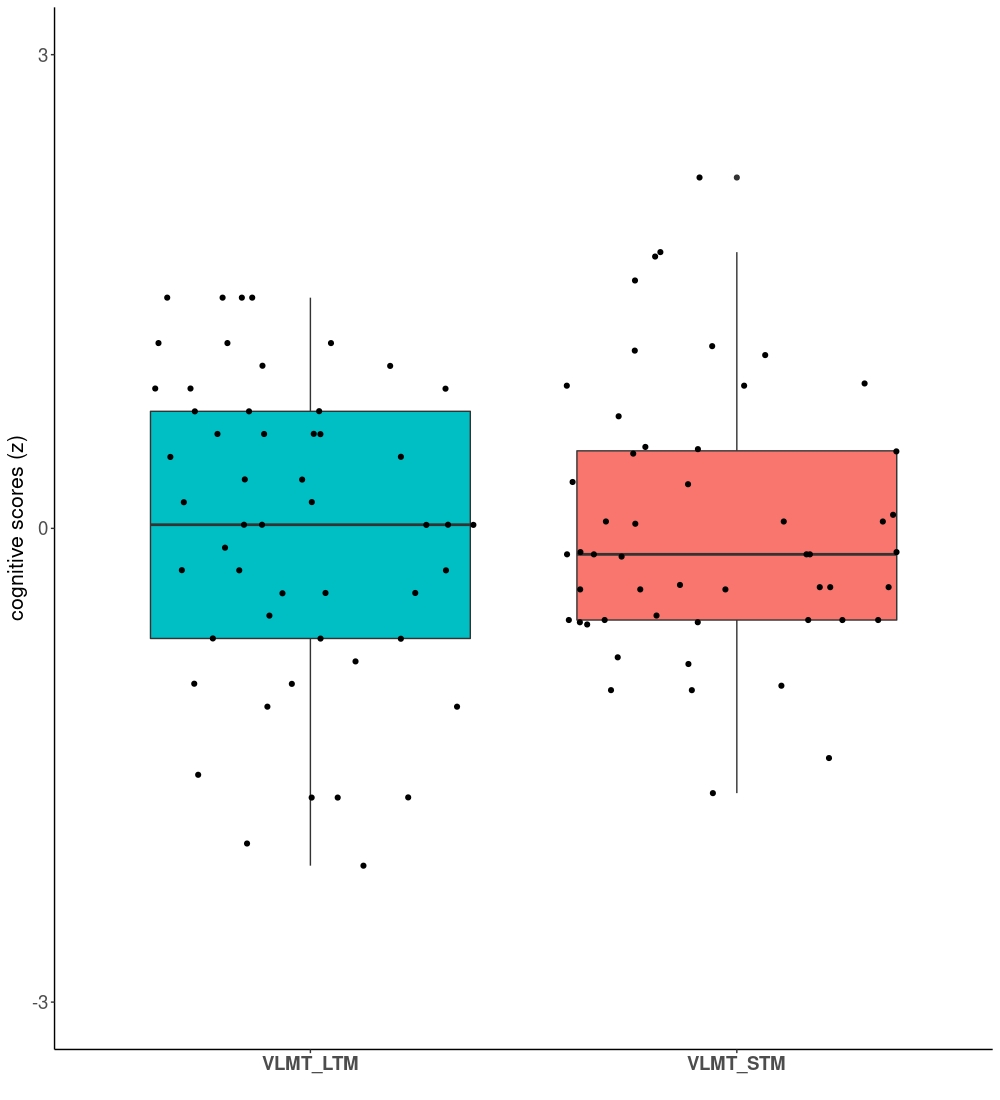


*Note.* The distributions of the z-standardized memory scores. The short-term memory score (VLMT_STM) represents the mean of the z-standardized scores in the first trial and the interference trial of the Verbal Learning and Memory Test (VLMT). The long-term memory score (VLMT_LTM) reflects the mean of the z-standardized scores in the sixth and seventh trials of the VLMT. No z-values were higher than 3 or lower than -3.

**Figure S5.2**

Distributions of the volumes of the hippocampal formation subfields


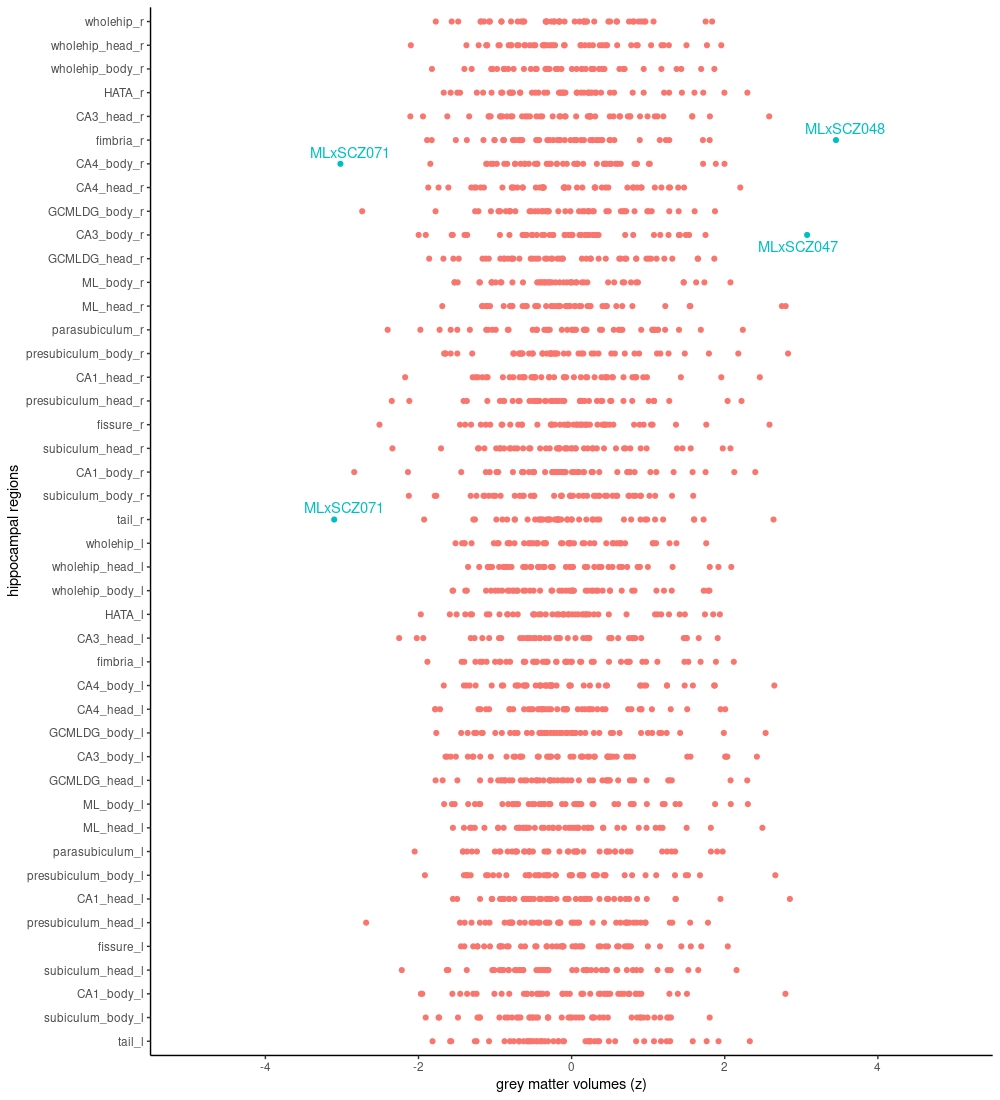


*Note.* The distributions of the z-standardized volumes of the hippocampal formation subfields. Z-values higher than 3 and lower than -3 are shown in turquoise and labelled with the corresponding participant identification code.

**Figure S5.3**

Functional connectivity between seeds from the hippocampus and middle frontal gyrus


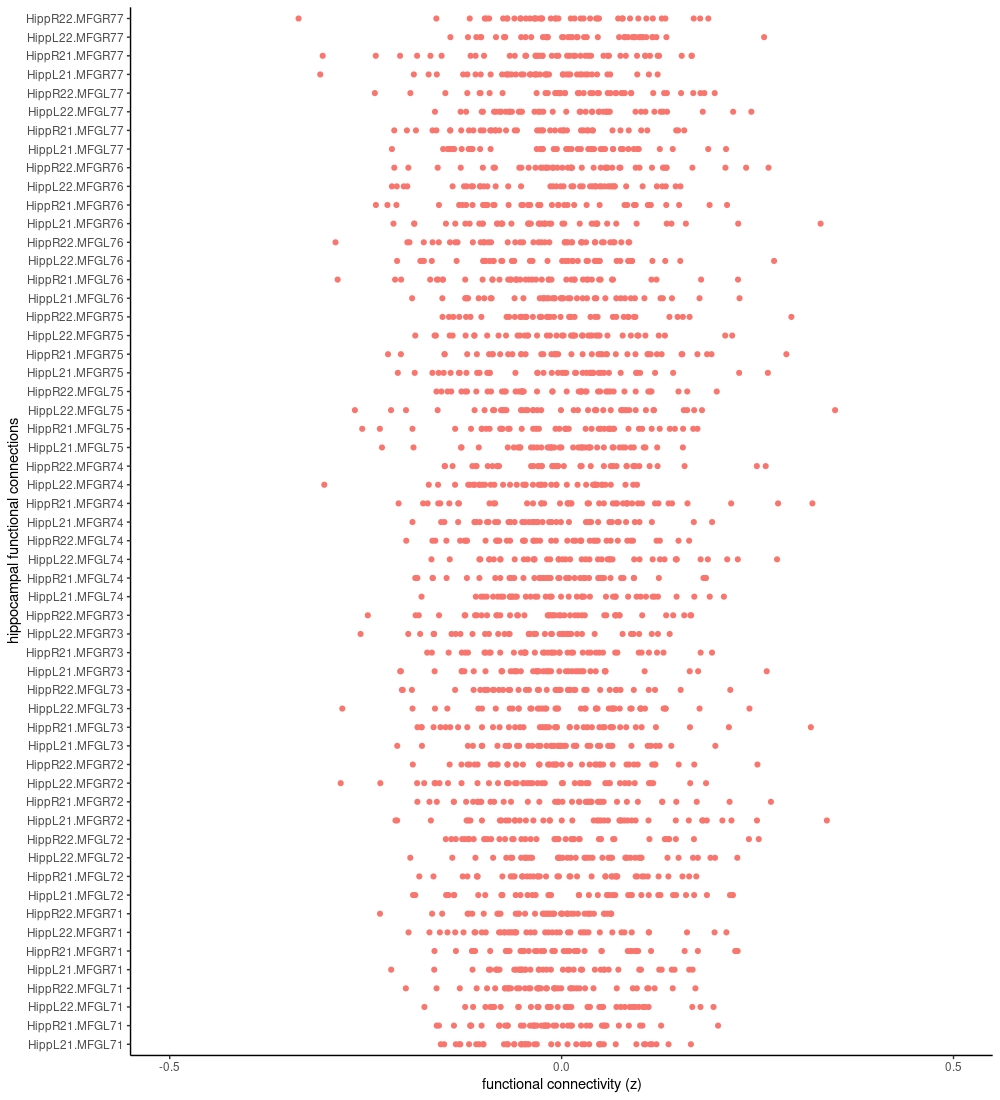


*Note.* The distributions of the z-standardized functional connectivity values between the hippocampus and the middle frontal gyrus. No z-values were higher than 3 or lower than -3.

**Figure S5.4**

Functional connectivity between seeds from the hippocampus, parahippocampal gyrus, and cingulate gyrus


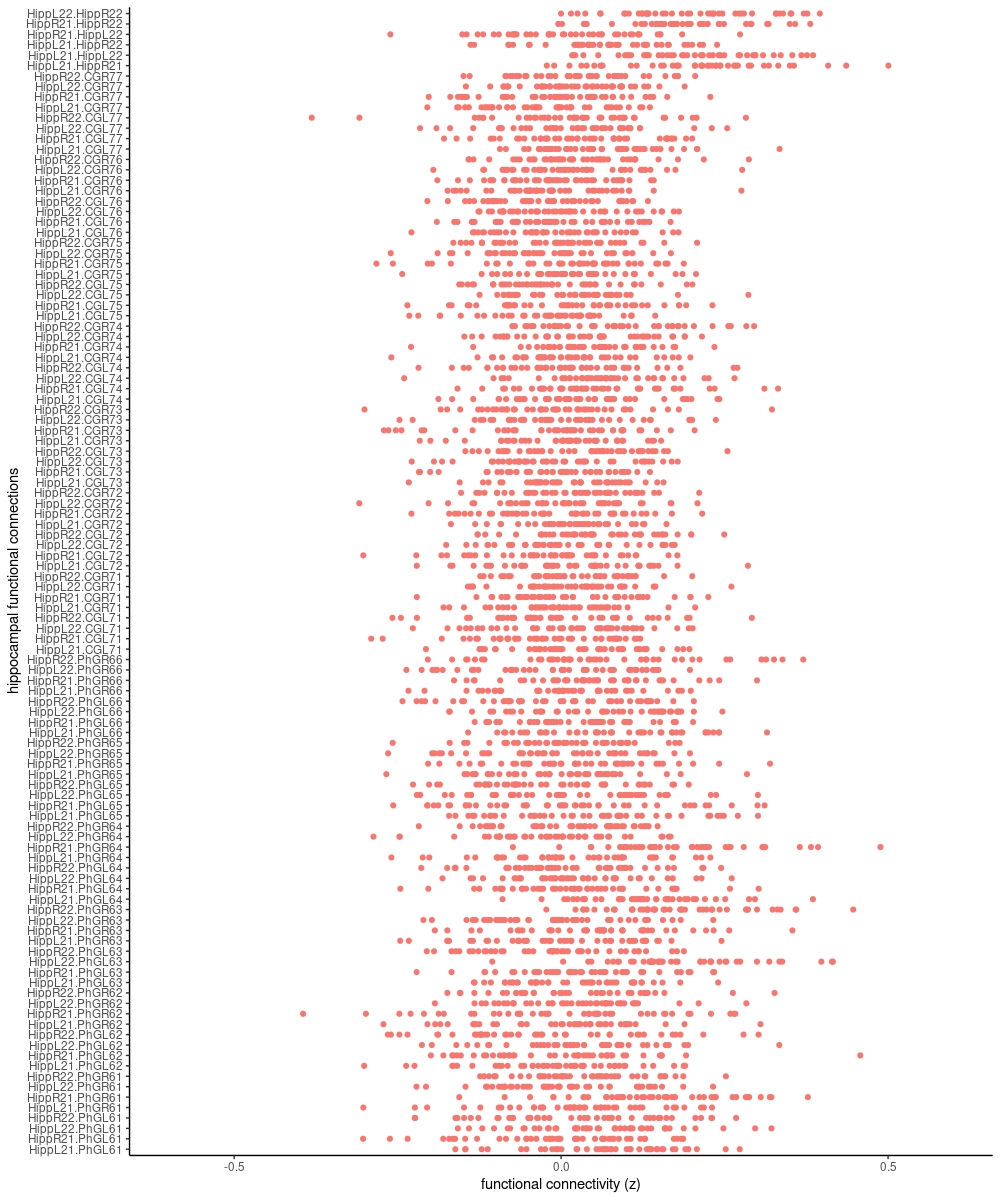


*Note.* The distributions of the z-standardized functional connectivity values between the hippocampus and the parahippocampal and cingulate gyrus. No z-values were higher than 3 or lower than -3.

**Tests of pre-assumptions in the multivariate multiple linear regression**

In multivariate multiple linear regression (MMLR), the following pre-assumptions have to be fulfilled: normal distribution, homoscedasticity, linearity, independence, and no multicollinearity. Regarding the assumption of normal distribution, Figures S5.1 to S5.4 illustrate the histogram plots based on the standardized residuals and the QQ plots for both the volume and functional connectivity data. A visual inspection indicates that the assumption of normal distribution is met because the histograms are distributed symmetrically around zero and points within the QQ plots build a straight linear line. Figures S5.5 and S5.6 show that homoscedasticity and linearity are given because the points are equally distributed around the regression line. The assumption of independence was confirmed by two non-significant Durbin-Watson (DW) tests for volume (DW = 2.00; p = 0.513) and functional connectivity data (DW = 2.01; p = 0.541). As indicated in Table S5, multicollinearity was precluded because the corresponding variance inflation factors were substantially lower than ten and the tolerance values were clearly higher than 0.2.

**Figure S6.1**

Normal distribution assumption for volume data: histogram plot


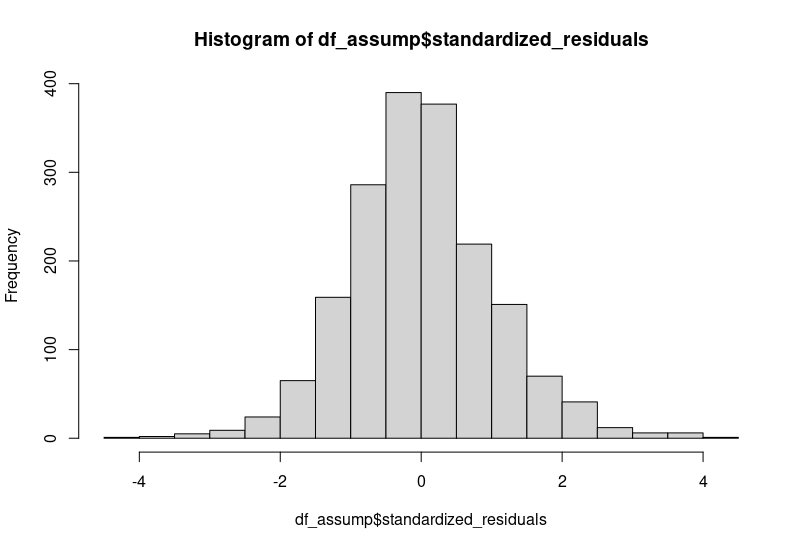


*Note.* The distribution of the standardized residuals of the multivariate multiple linear regression predicting the volumes in the hippocampal formation subfields.

**Figure S6.2**

Normal distribution assumption for volume data: QQ plot


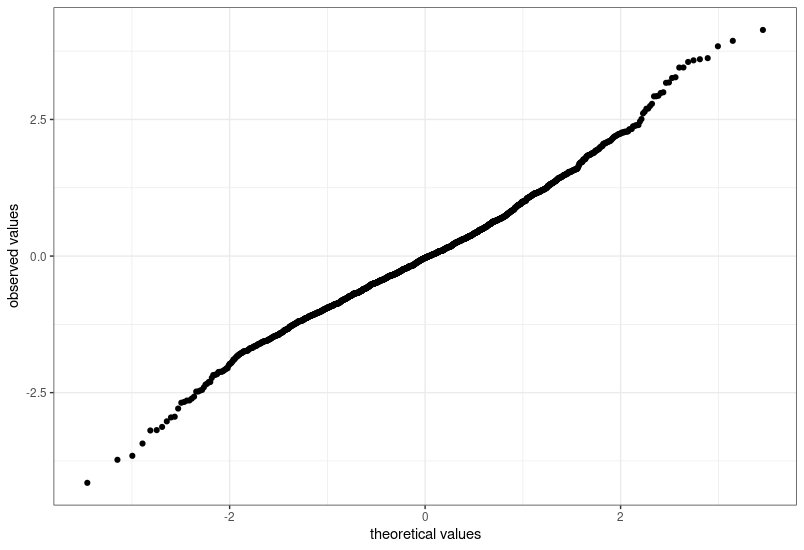


*Note.* The theoretical and observed values of the multivariate multiple linear regression predicting the volumes in the hippocampal formation subfields.

**Figure S6.3**

Normal distribution assumption for functional connectivity data: histogram plot


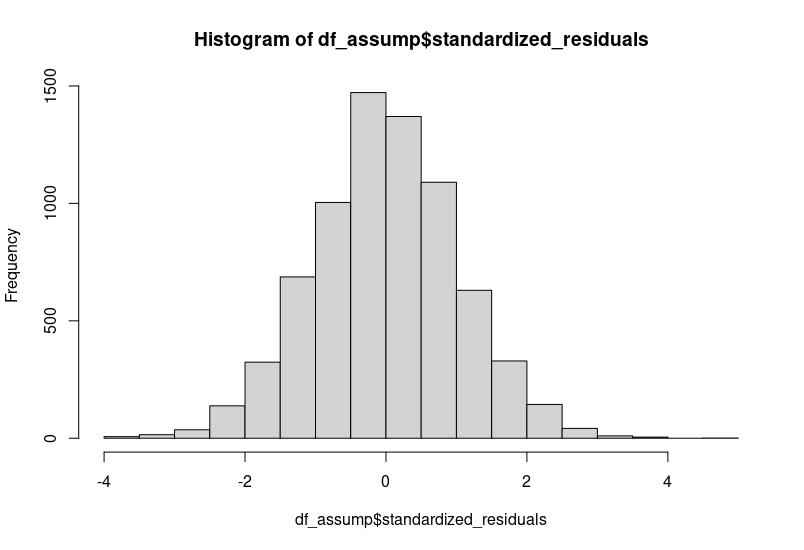


*Note.* The distribution of the standardized residuals of the multivariate multiple linear regression predicting hippocampal functional connectivity to the parahippocampal gyrus, middle frontal gyrus, and cingulate gyrus.

**Figure S6.4**

Normal distribution assumption for functional connectivity data: QQ plot


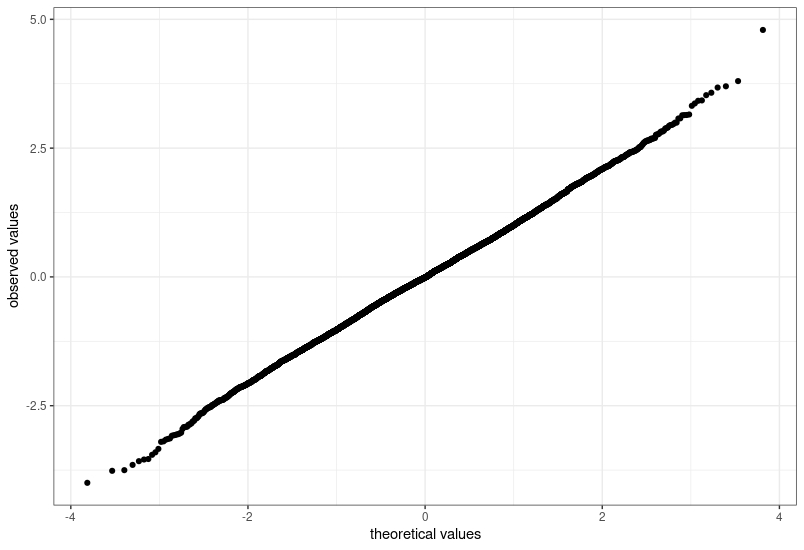


*Note.* The theoretical and observed values of the multivariate multiple linear regression predicting hippocampal functional connectivity to the parahippocampal gyrus, middle frontal gyrus, and cingulate gyrus.

**Figure S6.5**

Homoscedasticity and linearity assumption for volume data


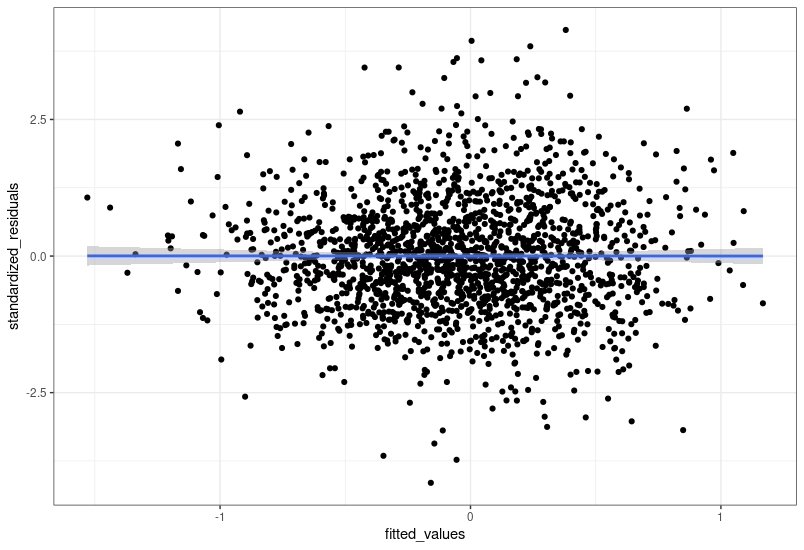


*Note.* The fitted values and standardized residuals of the MMLR predicting the volumes in the hippocampal formation subfields.

**Figure S6.6**

Homoscedasticity and linearity assumption for functional connectivity data


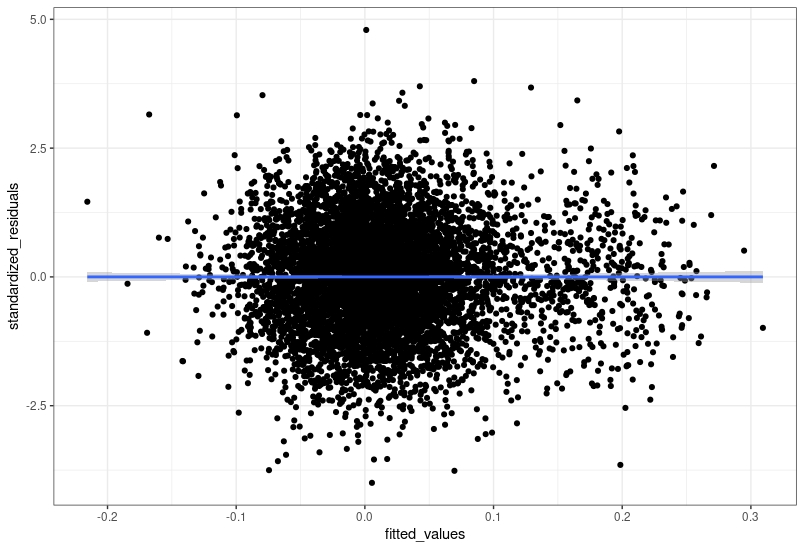


*Note.* The fitted values and standardized residuals of the multivariate multiple linear regression predicting hippocampal functional connectivity to the parahippocampal gyrus, middle frontal gyrus, and cingulate gyrus.

**Table S6**

Multicollinearity: variance inflation factors and tolerances

| **Data** | **Predictors** | **VIF** | **Tolerance** |
| --- | --- | --- | --- |
|  |  |  |  |
| Volumes | Aerobic fitness | 1.55 | 0.64 |
|  | BMI | 1.36 | 0.74 |
|  | Age | 1.91 | 0.52 |
|  | Sex | 1.16 | 0.86 |
|  | EY | 1.14 | 0.88 |
|  | DD | 1.60 | 0.63 |
|  | CPZ | 1.27 | 0.79 |
|  |  |  |  |
| Functional connectivity | Aerobic fitness | 1.66 | 0.60 |
|  | BMI | 1.38 | 0.72 |
|  | Age | 1.84 | 0.54 |
|  | Sex | 1.61 | 0.86 |
|  | EY | 1.13 | 0.88 |
|  | DD | 1.44 | 0.69 |
|  | CPZ | 1.12 | 0.90 |

*Note.* The variance inflation factors and corresponding tolerances within the two MMLR predicting the volumes of the hippocampal formation subfields and hippocampal functional connectivity to the parahippocampal gyrus, middle frontal gyrus and cingulate gyrus.

BMI, Body mass index; CPZ, chlorpromazine equivalents; DD, disorder duration; EY, education years; VIF, variance inflation factor

**Effect sizes in the MMLR on hippocampal functional connectivity**

Figures S6.1 and S6.2 display the β-coefficients of the MMLR predicting hippocampal functional connectivity to itself and to the parahippocampal, middle frontal, and cingulate gyrus.

**Figure S7.1**

β-coefficients of the multivariate multiple linear regression predicting hippocampal functional connectivity


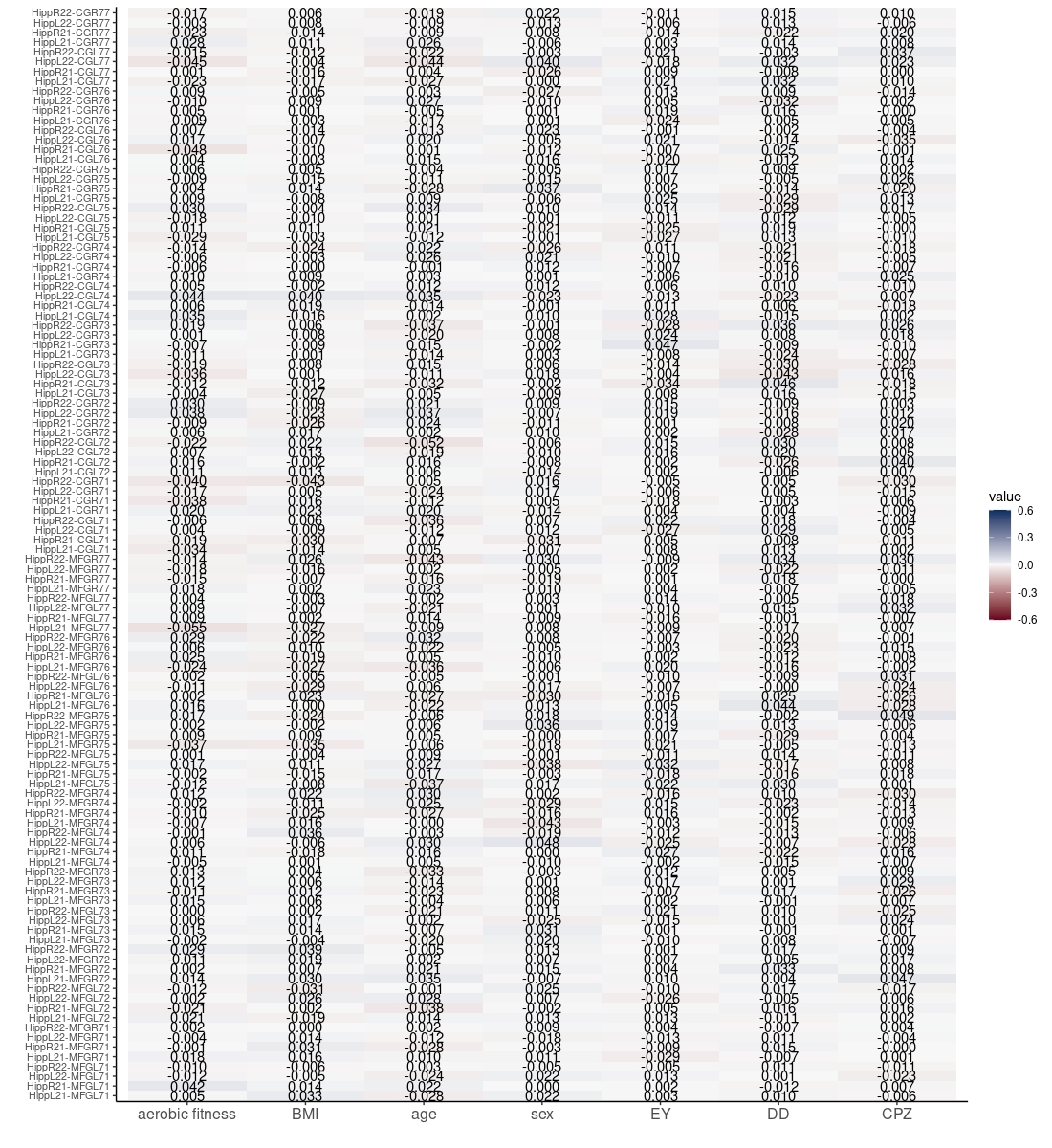


*Note.* The x-axis shows the predictors of the MMLR, and the y-axis, the functional connections between seeds from the hippocampus, cingulate gyrus, and middle frontal gyrus. The heatmap is filled with the β-coefficients. Blue indicates a positive effect, red a negative effect. The darker the color, the stronger is the effect in the corresponding direction.

BMI, Body mass index; CG, cingulate gyrus CPZ, chlorpromazine equivalents; DD, disorder duration; EY, education years; Hip, hippocampus;; MFG, middle frontals gyrus.

Details of the seed labels are described in the Brainnetome Atlas [30].

**Figure S7.2**

β-coefficients of the multivariate multiple linear regression predicting hippocampal functional connectivity


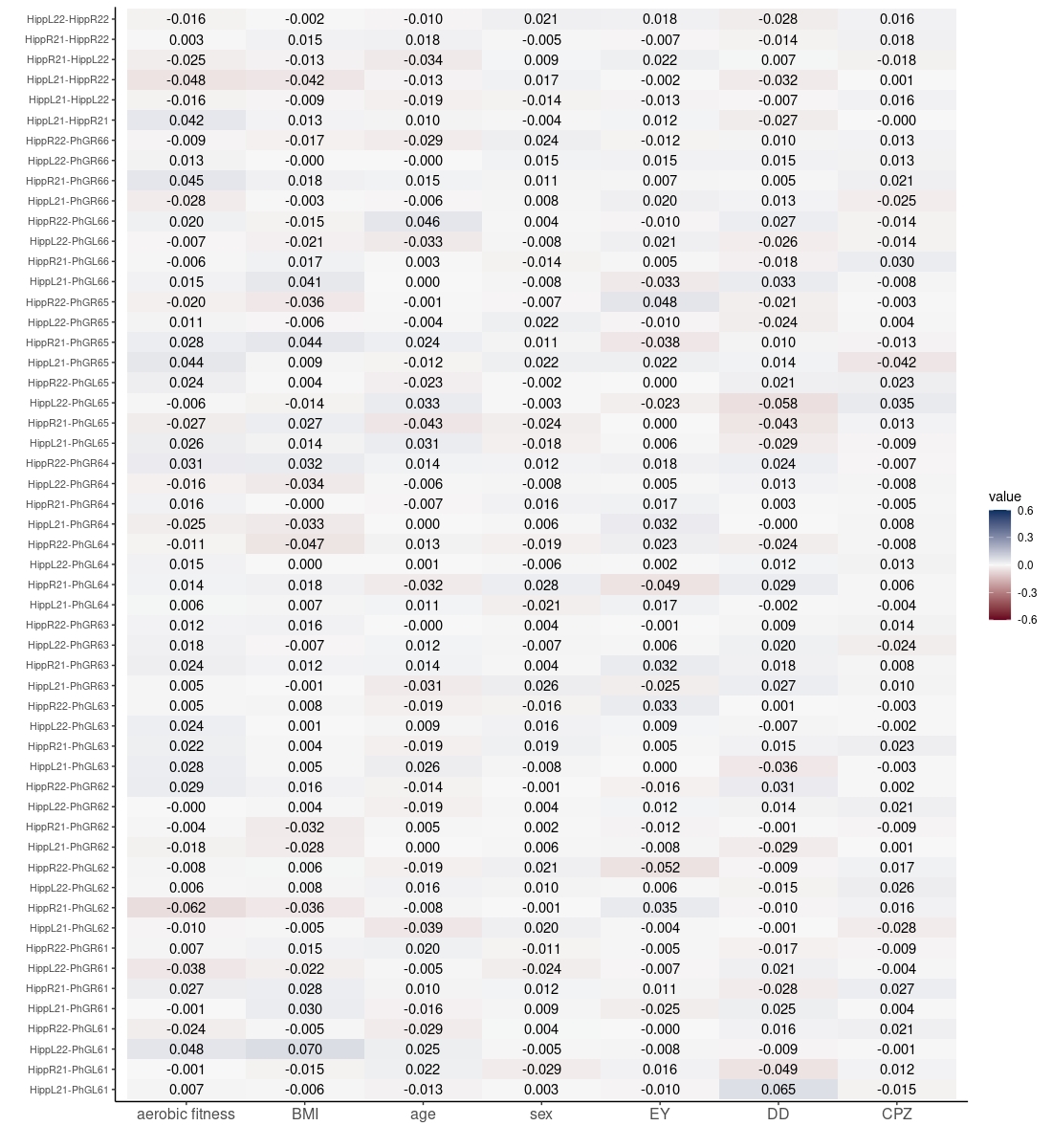


*Note.* The x-axis shows the predictors of the MMLR, and the y-axis, the functional connections between seeds from the hippocampus and between seeds from the hippocampus and parahippocampal gyrus. The heatmap is filled with the β-coefficients. Blue indicates a positive effect, red a negative effect. The darker the color, the stronger is the effect in the corresponding direction.

BMI, body mass index; CPZ, chlorpromazine equivalents; DD, disorder duration; EY, education years; Hip, hippocampus; PhG, parahippocampal gyrus

Details of the seed labels are described in the Brainnetome Atlas [30].

**Robustness checks of the MMLR**

Table S8.1 summarizes the results of the robustness checks of the MMLR predicting the volumes of the hippocampal formation subfields. We tested four alternative models and compared them with the model described in the main manuscript. These models differed regarding the type and number of included covariates. Across all models, aerobic fitness significantly predicted the volumes in the subfields of the hippocampal formation.

Table S8.2 presents the results from a different statistical approach that could have been used to examine the association between aerobic fitness and hippocampal formation subfield volumes. This approach calculated 38 multiple linear regressions with aerobic fitness and the covariates as predictors and the individual hippocampal formation subfield volumes as dependent variables. As shown in Table S8.2, in 16 of the 38 (42.1%) regression models, aerobic fitness was a significant predictor of the corresponding subfield volume. Assuming the null hypothesis and an alpha level of 0.05, we would have expected approximately two significant results (38 x 0.05 = 1.9). Consequently, at first glance the alternative statistical approach supports our finding from the main manuscript. However, after controlling for the false discovery rate [31], the associations between fitness and subfield volumes were no longer significant. Hence, we emphasize that the identified associations between aerobic fitness and the individual hippocampal formation subfield volumes are not completely robust when tested with strict statistical approaches and need further replication in an independent sample.

**Table S8.1**

Robustness checks of the multivariate multiple linear regression model predicting volumes of the hippocampal formation subfields

| **Model** | **Predictor** | **t(df)** | **R²_pseudo_** | **p** |
| --- | --- | --- | --- | --- |
|  |  |  |  |  |
| 1 | Omnibus effect | 1.7437469 (3) | 0.10625856 | **0.03112226*** |
|  | Aerobic fitness | 2.7370782 (1) | 0.05559637 | **0.01525618*** |
|  | Sex | 1.1750219 (1) | 0.0238674 | 0.28224002 |
|  | Age | 1.7264788 (1) | 0.03506877 | 0.09639406 |
|  |  |  |  |  |
| 2 | Omnibus effect | 1.77866 (4) | 0.14196730 | **0.016133924*** |
|  | Aerobic fitness | 3.6797999 (1) | 0.07342764 | **0.003093266*** |
|  | Sex | 1.195658 (1) | 0.02385846 | 0.271191296 |
|  | Age | 1.7184004 (1) | 0.03428939 | 0.097983116 |
|  | BMI | 1.7895306 (1) | 0.03570874 | 0.085549051 |
|  |  |  |  |  |
| 3 | Omnibus effect | 1.5063861 (5) | 0.15206212 | **0.045033598*** |
|  | Aerobic fitness | 3.7168156 (1) | 0.07503878 | **0.002918845*** |
|  | Sex | 1.1790398 (1) | 0.02380363 | 0.280261303 |
|  | Age | 1.573831 (1) | 0.03177407 | 0.129556569 |
|  | BMI | 1.6323168 (1) | 0.03295484 | 0.115708532 |
|  | EY | 0.5000161 (1) | 0.01009482 | 0.871122042 |
|  |  |  |  |  |
| 4 | Omnibus effect | 1.5241571 (6) | 0.18237019 | **0.031636745*** |
|  | Aerobic fitness | 3.6677063 (1) | 0.07314210 | **0.003175645*** |
|  | sex | 1.0844386 (1) | 0.02162608 | 0.336925045 |
|  | age | 1.5159891 (1) | 0.03023214 | 0.145066174 |
|  | BMI | 1.4984193 (1) | 0.02988176 | 0.150110947 |
|  | EY | 0.5084792 (1) | 0.01014019 | 0.86362351 |
|  | CPZ | 1.5197965 (1) | 0.03030807 | 0.143996262 |

*Note.* Four alternative multivariate multiple linear regression models to the model described in the main manuscript. Models differed regarding the included covariates.

Significant p values (< 0.05) are written in bold and marked with an asterisk.

BMI, body mass index; CPZ, chlorpromazine equivalents; EY, years of education; omnibus effect, effect of the whole MMLR model; R²_pseudo_, effect size similar to the coefficient of determination; t(df), test statistics with degrees of freedom

**Table S8.2**

Results based on an alternative statistical approach with separate multiple linear regressions for each subfield

| **Hippocampal formation subfield** | **Predictor** | **P** | **P_fdr_** |
| --- | --- | --- | --- |
|  |  |  |  |
| Tail-r | Aerobic fitness | 0.07377084 | 0.13431064 |
| Tail-l | Aerobic fitness | 0.1086584 | 0.15880845 |
| Subiculum-head-r | Aerobic fitness | **0.04181445*** | 0.1107215 |
| Subiculum-head-l | Aerobic fitness | 0.08427406 | 0.14327705 |
| Subiculum-body-r | Aerobic fitness | 0.4231321 | 0.50246932 |
| Subiculum-body-l | Aerobic fitness | 0.1280691 | 0.18024536 |
| Presubiculum-head-r | Aerobic fitness | **0.01203867*** | 0.07960208 |
| Presubiculum-head-l | Aerobic fitness | **0.01853124*** | 0.08802338 |
| Presubiculum-body-r | Aerobic fitness | 0.8829445 | 0.90680788 |
| Presubiculum-body-l | Aerobic fitness | 0.7905286 | 0.83444682 |
| Parasubiculum-r | Aerobic fitness | 0.0901494 | 0.14327705 |
| Parasubiculum-l | Aerobic fitness | 0.2951444 | 0.37628487 |
| ML-head-r | Aerobic fitness | **0.01466354*** | 0.07960208 |
| ML-head-l | Aerobic fitness | **0.02808*** | 0.09700364 |
| ML-body-r | Aerobic fitness | 0.5020407 | 0.5781075 |
| ML-body-l | Aerobic fitness | 0.7777846 | 0.83444682 |
| HATA-r | Aerobic fitness | 0.1026381 | 0.15600996 |
| HATA-l | Aerobic fitness | 0.297067 | 0.37628487 |
| GCMLDG-head-r | Aerobic fitness | **0.01302613*** | 0.07960208 |
| GCMLDG-head-l | Aerobic fitness | 0.06265928 | 0.1322807 |
| GCMLDG-body-r | Aerobic fitness | 0.09049077 | 0.14327705 |
| GCMLDG-body-l | Aerobic fitness | **0.03959119*** | 0.1107215 |
| Fissure-r | Aerobic fitness | 0.2618098 | 0.35531331 |
| Fissure-l | Aerobic fitness | 0.4223969 | 0.50246932 |
| Fimbria-r | Aerobic fitness | 0.9745618 | 0.97456184 |
| Fimbria-l | Aerobic fitness | 0.7705593 | 0.83444682 |
| CA4-head-r | Aerobic fitness | **0.002852028*** | 0.06946652 |
| CA4-head-l | Aerobic fitness | **0.02590933*** | 0.09700364 |
| CA4-body-r | Aerobic fitness | **0.03315269*** | 0.10498351 |
| CA4-body-l | Aerobic fitness | **0.008351368*** | 0.07960208 |
| CA3-head-r | Aerobic fitness | **0.003656133*** | 0.06946652 |
| CA3-head-l | Aerobic fitness | 0.0742243 | 0.13431064 |
| CA3-body-r | Aerobic fitness | **0.04930761*** | 0.11710558 |
| CA3-body-l | Aerobic fitness | **0.01253047*** | 0.07960208 |
| CA1-head-r | Aerobic fitness | **0.02214488*** | 0.09350061 |
| CA1-head-l | Aerobic fitness | **0.04370586*** | 0.1107215 |
| CA1-body-r | Aerobic fitness | 0.06656729 | 0.13313458 |
| CA1-body-l | Aerobic fitness | 0.06125699 | 0.1322807 |

*Note.* Significance of aerobic fitness in 38 separate multiple linear regressions with the hippocampal formation subfield volumes as dependent variables; 16 of the 38 regressions were significant at first but not after false discovery rate correction for multiple comparisons. Covariates in the multiple linear regression models were age, sex, body mass index, education years, disorder duration, and chlorpromazine equivalents.

Significant p values (< 0.05) are written in bold and marked with an asterisk.

**Post hoc power analyses**

We used the pwr package in R to compute two power analyses for our multiple regression models predicting the hippocampal formation subfield volumes and hippocampal functional connectivity. For the volumetric data, we achieved a statistical power of 30.0% with 48 participants and seven predictors and assuming a small effect size of f^2^ = 0.1 and a significance level of α = 0.05. For the functional data, we achieved a statistical power of 27.5% with 44 participants and seven predictors and assuming a small effect size of f^2^ = 0.1 and a significance level of α = 0.05.

We calculated an additional power analysis for the mediation models with the powerMediation package in R. Given a sample size of 47, a slope of β = 0.2, an SD of 1 for the mediator and 1 for the random error term, and a significance level of α = 0.05, we achieved a statistical power of 27.5%.

**References**

1. Reuter M, Rosas HD, Fischl B. Highly accurate inverse consistent registration: a robust approach. *Neuroimage* 2010; 53(4)**:** 1181-1196.

2. Ségonne F, Dale AM, Busa E, Glessner M, Salat D, Hahn HK *et al.* A hybrid approach to the skull stripping problem in MRI. *Neuroimage* 2004; 22(3)**:** 1060-1075.

3. Fischl B, Salat DH, Busa E, Albert M, Dieterich M, Haselgrove C *et al.* Whole brain segmentation: automated labeling of neuroanatomical structures in the human brain. *Neuron* 2002; 33(3)**:** 341-355.

4. Fischl B, Salat DH, van der Kouwe AJ, Makris N, Ségonne F, Quinn BT *et al.* Sequence-independent segmentation of magnetic resonance images. *Neuroimage* 2004; 23 Suppl 1**:** S69-84.

5. Sled JG, Zijdenbos AP, Evans AC. A nonparametric method for automatic correction of intensity nonuniformity in MRI data. *IEEE Trans Med Imaging* 1998; 17(1)**:** 87-97.

6. Fischl B, Liu A, Dale AM. Automated manifold surgery: constructing geometrically accurate and topologically correct models of the human cerebral cortex. *IEEE Trans Med Imaging* 2001; 20(1)**:** 70-80.

7. Ségonne F, Pacheco J, Fischl B. Geometrically accurate topology-correction of cortical surfaces using nonseparating loops. *IEEE Trans Med Imaging* 2007; 26(4)**:** 518-529.

8. Dale AM, Fischl B, Sereno MI. Cortical surface-based analysis. I. Segmentation and surface reconstruction. *Neuroimage* 1999; 9(2)**:** 179-194.

9. Dale AM, Sereno MI. Improved Localizadon of Cortical Activity by Combining EEG and MEG with MRI Cortical Surface Reconstruction: A Linear Approach. *J Cogn Neurosci* 1993; 5(2)**:** 162-176.

10. Fischl B, Dale AM. Measuring the thickness of the human cerebral cortex from magnetic resonance images. *Proc Natl Acad Sci U S A* 2000; 97(20)**:** 11050-11055.

11. Tustison NJ, Avants BB, Cook PA, Zheng Y, Egan A, Yushkevich PA *et al.* N4ITK: improved N3 bias correction. *IEEE Trans Med Imaging* 2010; 29(6)**:** 1310-1320.

12. Avants BB, Tustison N, Song G. Advanced normalization tools (ANTS). *Insight j* 2009; 2(365)**:** 1-35.

13. Fonov VS, Evans AC, McKinstry RC, Almli CR, Collins DL. Unbiased nonlinear average age-appropriate brain templates from birth to adulthood. *NeuroImage* 2009; 47**:** 102.

14. Avants BB, Epstein CL, Grossman M, Gee JC. Symmetric diffeomorphic image registration with cross-correlation: Evaluating automated labeling of elderly and neurodegenerative brain. *Med Image Anal* 2008; 12(1)**:** 26-41.

15. Zhang Y, Brady M, Smith S. Segmentation of brain MR images through a hidden Markov random field model and the expectation-maximization algorithm. *IEEE Transactions on Medical Imaging* 2001; 20(1)**:** 45-57.

16. Jenkinson M, Beckmann CF, Behrens TE, Woolrich MW, Smith SM. FSL. *NeuroImage* 2012; 62(2)**:** 782-790.

17. Cox RW. AFNI: Software for Analysis and Visualization of Functional Magnetic Resonance Neuroimages. *Computers and Biomedical Research* 1996; 29(3)**:** 162-173.

18. Jenkinson M, Bannister P, Brady M, Smith S. Improved Optimization for the Robust and Accurate Linear Registration and Motion Correction of Brain Images. *NeuroImage* 2002; 17(2)**:** 825-841.

19. Wang S, Peterson DJ, Gatenby JC, Li W, Grabowski TJ, Madhyastha TM. Evaluation of Field Map and Nonlinear Registration Methods for Correction of Susceptibility Artifacts in Diffusion MRI. *Front Neuroinform* 2017; 11**:** 17.

20. Huntenburg JM, Gorgolewski KJ, Anwander A, Margulies DS. Evaluating nonlinear coregistration of BOLD EPI and T1 images. *F1000 Res* 2014; 5**:** 740.

21. Treiber JM, White NS, Steed TC, Bartsch H, Holland D, Farid N *et al.* Characterization and Correction of Geometric Distortions in 814 Diffusion Weighted Images. *PLOS ONE* 2016; 11(3)**:** e0152472.

22. Greve DN, Fischl B. Accurate and robust brain image alignment using boundary-based registration. *NeuroImage* 2009; 48(1)**:** 63-72.

23. Jenkinson M, Smith S. A global optimisation method for robust affine registration of brain images. *Med Image Anal* 2001; 5(2)**:** 143-156.

24. Power JD, Mitra A, Laumann TO, Snyder AZ, Schlaggar BL, Petersen SE. Methods to detect, characterize, and remove motion artifact in resting state fMRI. *NeuroImage* 2014; 84**:** 320-341.

25. Gorgolewski K, Burns CD, Madison C, Clark D, Halchenko YO, Waskom ML *et al.* Nipype: a flexible, lightweight and extensible neuroimaging data processing framework in python. *Front Neuroinform* 2011; 5**:** 13.

26. Pruim RHR, Mennes M, van Rooij D, Llera A, Buitelaar JK, Beckmann CF. ICA-AROMA: A robust ICA-based strategy for removing motion artifacts from fMRI data. *NeuroImage* 2015; 112**:** 267-277.

27. Esteban O, Birman D, Schaer M, Koyejo OO, Poldrack RA, Gorgolewski KJ. MRIQC: Advancing the automatic prediction of image quality in MRI from unseen sites. *PLoS One* 2017; 12(9)**:** e0184661.

28. Raamana P, Theyers A, Selliah T, Bhati P, Arnott S, Hassel S *et al.* Visual QC Protocol for FreeSurfer Cortical Parcellations from Anatomical MRI. 2020.

29. Esteban O, Markiewicz CJ, Blair RW, Moodie CA, Isik AI, Erramuzpe A *et al.* fMRIPrep: a robust preprocessing pipeline for functional MRI. *Nat Methods* 2019; 16(1)**:** 111-116.

30. Fan L, Li H, Zhuo J, Zhang Y, Wang J, Chen L *et al.* The Human Brainnetome Atlas: A New Brain Atlas Based on Connectional Architecture. *Cerebral Cortex* 2016; 26(8)**:** 3508-3526.

31. Benjamini Y, Hochberg Y. Controlling the False Discovery Rate: A Practical and Powerful Approach to Multiple Testing. *Journal of the Royal Statistical Society* 1995; 57(1)**:** 289-300.
